# Three Rickettsioses, Darnley Island, Australia

**DOI:** 10.3201/eid1307.050088

**Published:** 2007-07

**Authors:** Nathan B. Unsworth, John Stenos, Antony G. Faa, Stephen R. Graves

**Affiliations:** * The Australian Rickettsial Reference Laboratory, Geelong, Victoria, Australia; † Warwick Hospital, Southern Downs Health Services District, Warwick, Queensland, Australia

**Keywords:** Torres Strait islands, Queensland tick typhus, scrub typhus, Rickettsia australis, Orientia tsutsugamushi, dispatch

## Abstract

We report 3 rickettsioses on Darnley Island, Australia, in the Torres Strait. In addition to previously described cases of Flinders Island spotted fever (*Rickettsia honei* strain “marmionii”), we describe 1 case of Queensland tick typhus (*R. australis*) and 2 cases of scrub typhus caused by a unique strain (*Orientia tsutsugamushi*).

The Torres Strait islands are scattered between Cape York in northeastern Australia and Papua New Guinea. Darnley (Erub) Island is the largest island (5.7 km^2^) in the eastern Torres Strait and has a mostly indigenous population of 360.

Rickettsioses in northeastern Australia include Queensland tick typhus (*Rickettsia australis*) (*1*,*2*), murine typhus (*R. typhi*), scrub typhus (*Orientia tsutsugamushi*) (*3*), and Flinders Island spotted fever (*R. honei* strain “marmionii”) (*4*). The latter 2 diseases are endemic to the Torres Strait islands (*4*,*5*). Because all 4 diseases have similar clinical manifestations, which may include maculopapular rash, fever, headache, rigor, myalgia, and arthralgia (*1**–**4*), laboratory investigation is needed to identify the rickettsial etiologic agent. We describe the northernmost case of Queensland tick typhus and 2 cases of scrub typhus, along with their molecular identifications, from patients examined at the Darnley Island Health Clinic.

## The Cases

In March 2003, a 23-year-old man (patient 1) sought treatment for fever (39.3°C), headache, and an eschar on his right thigh. He had no rash. No diagnosis was made, but he was given penicillin V. Seven days later he returned with worsening symptoms of fever (39.9°C), headache, cough, arthralgia, and lethargy. A provisional diagnosis of scrub typhus was made, and he was given doxycycline. By the next day he was afebrile, and his condition was much improved. The diagnosis was confirmed by a positive serologic result for scrub typhus rickettsiae (antibody titer 512) and the presence of *O. tsutsugamushi* in blood detected on day 7 by PCR and culture (Table).

**Table:** Rickettsial detection, Darnley Island, Australia, 2003–2004

Patient	Serum sample no. 1			Serum sample no. 2			*Orientia tsutsugamushi*		*Rickettsia australis*	
	Days after disease onset	SFG titer*	STG titer†	Days after disease onset	SFG titer*	STG titer†				
							PCR	Culture	PCR	Culture
1	7	128	512	170	128	>1,024	+	+	–	–
2	10	128	<128	33	128	>1,024	+	+	–	–
3	3	256	<128	133	256	<128	–	–	–	+

*SFG, spotted fever group; antigens were *R. honei*, *R. australis*, *R. akari*, *R. conorii*, *R. siberica,* and *R. rickettsii.* †STG, scrub typus group; antigens were the *O. tsutsugamushi* strains Gilliam and Kato.

In May 2004, an 11-year-old boy (patient 2) was examined because of a 3-day history of fever (39.0°C), headache, nausea, abdominal pain, and a boil surrounded by cellulitis behind his left ear. He had no rash. He was given flucloxacillin for the boil. On day 10 he was still ill and febrile. His blood was tested for malaria, dengue, and scrub typhus, but he was given no antimicrobial therapy. He was not seen again until day 18, when he was prescribed doxycycline after PCR detected *O. tsutsugamushi* (and it was later isolated) in his day-10 blood sample. Follow-up serologic testing of samples collected on days 10 and 33 displayed seroconversion to antibodies against scrub typhus (titers <128 to >1,024; Table).

In April 2003, a 29-year-old man (patient 3) sought treatment for fever (38.4°C), cough, nausea, lethargy, and cellulitis of both feet. His left thigh had 2 boils, and his inguinal nodes were palpable. Three days later, he was evacuated by air and admitted to the Thursday Island hospital, at which time he was still febrile (39.2°C) but had no cellulitis. He also had a macular rash, which he reported as originally having been pustular; the thigh lesions were recognized as eschars. A tentative diagnosis of scrub typhus was made, and the patient was given doxycycline. Within 48 hours he was afebrile and discharged. Serologic testing for rickettsiae was positive for the spotted fever group (titer 256) on day 3, but the titers remained unchanged 4 months later. A spotted fever group rickettsial organism was grown from the day-3 blood sample; however, PCR was negative for rickettsiae (Table).

Rickettsial serologic testing was performed on patients’ paired serum specimens by using an indirect immunofluorescence assay (*6*). Titers >128 were deemed positive. Patient 1 had an increase in titer to *O. tsutsugamushi;* patient 2 exhibited seroconversion to *O. tsutsugamushi* antibodies; and patient 3 had stationary positive titers to spotted fever group rickettsiae (Table).

Rickettsial isolation was performed according to previously described methods (*7*). A spotted fever group rickettsial organism was isolated from patient 3, and an *Orientia* organism was isolated from patients 1 and 2 (Table). Only 1 *Orientia* organism could be adapted to continuous culture (patient 2).

We extracted DNA from enriched buffy coat and rickettsial cultures by using the QIAamp Blood Mini Kit (QIAGEN, Hilden, Germany) and following the manufacturer’s protocols. Scrub typhus was diagnosed by 56-kDa gene PCR, which used the primers A (5′-TACATTAGCTGCAGGTATGACA-3′) and B (5′-CCAGCATAATTCTTTAACCAAG-3′) (Invitrogen, Mount Waverley, Victoria, Australia) as previously described, without the nested procedure and with a 51°C annealing temperature (*8*). Buffy coats and cultures from patients 1 and 2 were PCR positive for *O. tsutsugamushi* (Table). The 320-bp product (patient 2) was sequenced (Newcastle DNA, University of Newcastle, Australia; GenBank accession no. AY860955) and shared 89.8% homology with the Taiwanese strains TW381 and TW521 (GenBank accession nos. AY222635 and AY222630, respectively). A phylogenetic tree of the 56-kDa antigen gene was constructed by using the SEQBOOT and CONSENSE programs of the PHYLIP software package (Figure).

**Figure:**
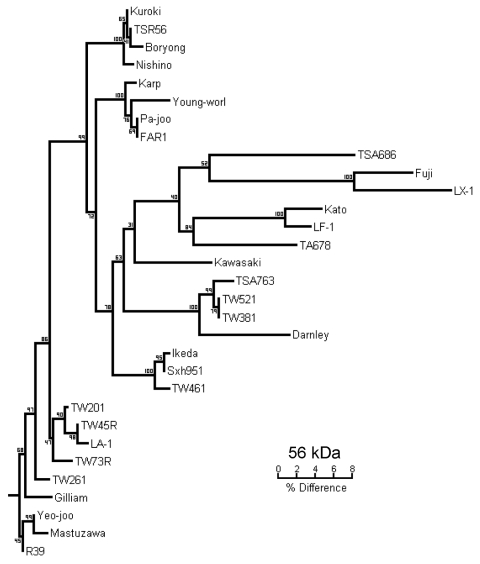
Phylogenetic tree obtained by a neighbor-joining analysis of the 56-kDa gene of *Orientia tsutsugamushi.* Bootstrap values from 100 analyses are shown at the node of each branch.

Spotted fever group rickettsemia was identified by 17-kDa antigen gene PCR that used the primers MTO-1 (5′-GCTCTTGCAACTCTATGTT-3′) and MTO-2 (5′-CATTGTTCGTCAGGTTGGCG-3′) (Invitrogen) as previously described, with an annealing temperature of 51°C and 45 cycles (*9*). The 17-kDa buffy coat PCR result was negative for patient 3, but the culture gave a 413-bp sequence that was 100% homologous with *R. australis* (GenBank accession no. M74042; Table). The patient’s buffy coat DNA extract was not tested for PCR inhibitors, and no attempt was made to use the *R. australis* isolate in a heterologous serologic reaction because the strain could not be established in continuous culture.

## Conclusions

Isolation of *R. australis* from a patient on Darnley Island redefines the northern limit of distribution of Queensland tick typhus in Australia. Previously, Queensland tick typhus had been thought to extend from Wilson’s Promontory (the tip of southeastern Australia) (*10*) to the Atherton Tableland (north Queensland) (*2*). This more northern finding of Queensland tick typhus was not unexpected because distribution of the vector of Queensland tick typhus in northeastern Australia, *Ixodes holocyclus*, is likely to include the Torres Strait islands and Papua New Guinea (*11*).

Rickettsial diseases that differ clinically from scrub typhus have been reported in Papua New Guinea (*12*). A recent serologic survey found 7 (3.7%) of 191 Papua New Guineans were seropositive to scrub typhus or spotted fever group rickettsiae (*13*). Another survey found that at least 19 (17%) of 113 Papua New Guineans had an antibody titer >256 against spotted fever group rickettsiae (A.G. Faa, unpub. data). A spotted fever group rickettsial disease such as Queensland tick typhus, Flinders Island spotted fever, or another undescribed rickettsiosis could explain these findings.

Low antibody titers (128) to the spotted fever group in the scrub typhus patients (Table) are consistent with previous exposure to spotted fever group or typhus group rickettsiae. Because 20% of each enriched buffy coat specimen was examined by rickettsial PCR and 80% by culture, results were skewed in favor of isolation rather than DNA detection.

Since 1935, numerous cases of scrub typhus have been reported in Australia and Papua New Guinea (*5*,*12*). Scrub typhus is known to be endemic to Darnley Island; however, strains have not been typed (*5*). Strains from northeastern Australia were serologically determined to be Karp or Karp-related (*14*). However, this new strain is 10.2% divergent from any other described strain, including Karp (Figure). Hence, we designated it as the Darnley strain, after the island from which it was isolated. The phylogenetic relationship of the Darnley strain to other Australian strains, including Litchfield, needs to be elucidated. The presence of 3 rickettsial diseases on this small island demonstrates the complexity of rickettsial epidemiology in Australia.
